# Differential Effects of Salient Visual Events on Memory‐Guided Attention in Adults and Children

**DOI:** 10.1111/cdev.13149

**Published:** 2018-10-08

**Authors:** Kate Nussenbaum, Gaia Scerif, Anna C. Nobre

**Affiliations:** ^1^ University of Oxford

## Abstract

Both salient visual events and scene‐based memories can influence attention, but it is unclear how they interact in children and adults. In Experiment 1, children (*N* = 27; ages 7–12) were faster to discriminate targets when they appeared at the same versus different location as they had previously learned or as a salient visual event. In contrast, adults (*N* = 30; ages 18–31) responded faster only when cued by visual events. While Experiment 2 confirmed that adults (*N* = 27) can use memories to orient attention, Experiment 3 showed that, even in the absence of visual events, the effects of memories on attention were larger in children (*N* = 27) versus adults (*N* = 28). These findings suggest that memories may be a robust source of influence on children's attention.

As we explore our cluttered world throughout childhood and adulthood, multiple sources influence where we orient our attention. Salient external events engage orienting mechanisms (e.g., Nobre, 2018; Posner, 1980; Posner & Peterson, 1990), facilitating our ability to detect or discriminate novel objects in our environments (Henderson, 1991; Müller & Rabbitt, 1989). Past experiences and their associated spatial memories also orient attention to relevant information (e.g., Giesbrecht, Sy, & Guerin, 2013; Goldfarb, Chun, & Phelps, 2016; Merrill, Conners, Roskos, Klinger, & Klinger, 2013; Rosen, Stern, & Somers, 2014; Rosenbaum & Jiang, 2013; Summerfield, Lepsien, Gitelman, Mesulam, & Nobre, 2006). Despite extensive research examining these two sources of orienting, the extent to which they *interact* in their influence on where children and adults orient their attention remains unclear.

Laboratory studies examining orienting responses to sudden visual events typically present those events on neutral backgrounds (e.g., Posner, 1980). However, as we explore and learn from the world around us, we frequently encounter the same environments over and over again—children spend the majority of the year within a single classroom; adults pass countless hours within their homes or workplaces. In the real world, the transient visual events that influence attention orienting in the lab are often present in environments with which we have extensive previous experience. For example, a child might learn that important information is typically written on the white board at the front of her classroom, causing her to orient more quickly to the board upon entering the room. But it is not clear how her experiences in learning to attend to the board at the front of her class might influence her attentional response to a novel, potentially distracting occurrence, like a classmate making silly faces in the seat next to her. It is important to understand, therefore, how salient events and our memories compete or cooperate to orient our attention to guide adaptive behavior. It may be the case that we use memories to orient attention only when no novel items appear in our environments. Alternatively, robust memories may cause us to ignore salient events toward which we would otherwise shift our attention. Critically, the extent to which sudden visual events or memories orient our attention may change over time as the cognitive and neural systems supporting memory encoding and spatial orienting develop. Understanding how novel visual events *that occur in familiar contexts* influence attention is key to understanding how memory and physical salience operate together in real‐world settings over developmental time.

## The Effects of Salient Visual Events on Attention Orienting Over Development

Posner's (1980) spatial cueing paradigm has been central to investigating the effects of salient visual events on the orienting of visuospatial attention. In one version of this paradigm, participants fixate centrally but are presented with a brief peripheral stimulus. After a short delay, they must detect or discriminate a target presented in either the cued location or a different, un‐cued, location (Posner, 1980; Posner & Cohen, 1984). From 4 months of age, infants look toward the cued targets more often and with greater speed relative to targets presented at un‐cued locations, demonstrating facilitated attention in response to external events (Johnson, Posner, & Rothbart, 1991; Johnson, 1994). The initial peripheral stimuli trigger *exogenous* orienting, so that subsequent stimuli presented at the cued location are prioritized for sensory processing (Posner, 1980).

Exogenous orienting responses to peripheral events remain robust throughout childhood (Iarocci, Enns, Randolph, & Burack, 2009; Schul, Townsend, & Stiles, 2003). Like infants, children demonstrate facilitated detection of targets that are presented at cued relative to un‐cued locations (Akhtar & Enns, 1989; Enns & Brodeur, 1989; Iarocci et al., 2009; Pozuelos, Paz‐Alonso, Castillo, Fuentes, & Rueda, 2014; Wainwright & Bryson, 2002). As they develop, children show a general increase in the speed of their attentional responses (Iarocci et al., 2009; Schul et al., 2003) and an enhanced ability to reorient to new locations (Schul et al., 2003). Despite these developmental improvements, individuals across age groups demonstrate consistent accuracy and reaction‐time benefits from being cued to the location of upcoming targets (Enns & Brodeur, 1989; Iarocci et al., 2009; Schul et al., 2003; Wainwright & Bryson, 2002).

## The Effects of Memories on Attention Orienting

A growing body of literature has also established memories as a critical influence on attention. Contextual cuing experiments provided convincing early laboratory demonstrations of these effects. In these experiments, individuals search through visual arrays to find a particular target shape or letter (Chun, 2000). Unbeknownst to them, over the course of the task, some of these arrays repeat. When these repeated arrays are interspersed with novel arrays, participants are faster to identify targets among the repeated arrays (Chun & Jiang, 1998). The effects of contextual cuing are robust: They persist when the location of the target is cued by the layout of the distractors and when the identity of the target is cued by the identity of the co‐occurring distractors (Chun, 2000).

Complementing contextual cuing effects, evidence from memory‐based orienting tasks also shows that scene‐based spatial memories influence attention. In these tasks, participants learn to associate scenes with specific spatial locations, either through repeatedly searching through the same scenes for objects hidden in a target location (Patai, Doallo, & Nobre, 2012; Salvato, Patai, & Nobre, 2016; Stokes, Atherton, Patai, & Nobre, 2012; Summerfield et al., 2006; Summerfield, Rao, Garside, & Nobre, 2011), or through identifying a subtle, localized change in the scene over several repetitions (Rosen, Stern, Michalka, Devaney, & Somers, 2015, 2016; Rosen et al., 2014). When these scenes act as cues in a subsequent orienting task, they yield enhanced responses (Patai et al., 2012; Rosen et al., 2016, 2014; Salvato et al., 2016; Stokes et al., 2012; Summerfield et al., 2006, 2011) to targets appearing at the location associated with the scene relative to other, un‐cued locations.

## Developmental Hypotheses

Unlike the robust effects of salient visual events on orienting (e.g., Schul et al., 2003), children's use of memories to guide attention is more heavily debated, and has been probed only through contextual cuing tasks (Barnes et al., 2008; Barnes, Howard, Howard, Kenealy, & Vaidya, 2010; Couperus, Hunt, Nelson, & Thomas, 2011; Dixon, Zelazo, & De Rosa, 2010; Merrill et al., 2013). Some work suggests that the mechanisms that enable memories to guide attention may emerge as early as infancy. Infants as young as 8 months can learn to associate specific contexts with the location of a hidden target (Bertels, San Anton, Gebuis, & Destrebecqz, 2016) and can use these associations to guide search for targets hidden in simple arrays (Tummeltshammer & Amso, 2017). These effects may persist into childhood, with children as young as 5 years old demonstrating effects of memories on attention (Dixon et al., 2010). But other evidence for children's use of memory‐guided attention is mixed (Vaidya, Huger, Howard, & Howard, 2007). Many factors, such as the number and nature of the distractors (Couperus et al., 2011), and the overall length of the task (Darby, Burling, & Yoshida, 2014), may contribute to whether children demonstrate contextual cuing effects. These mixed results suggest that children *can* use memories to guide attention, but that their ability to do so emerges only under optimal task conditions.

Neurodevelopmental data further explain why children's use of memories to guide attention may not be as robust as that of adults’. Previous work has implicated the involvement and coordination of medial temporal areas involved in memory recall and dorsal fronto‐parietal areas involved in attention orienting (Giesbrecht et al., 2013; Goldfarb et al., 2016; Rosen et al., 2016; Stokes et al., 2012; Summerfield et al., 2006). The flexible engagement of these two networks has been shown to be mediated by cognitive control mechanisms in the prefrontal cortex (Rosen et al., 2016)—mechanisms that continue to develop throughout the school‐aged years (Johnson, Munro, & Bunge, 2014). The relative immaturity of children's cognitive control systems, coupled with the mixed evidence for the presence of a contextual cuing effect in school‐aged children, suggests that the interactions between memory systems and orienting systems necessary to enable past experiences to guide attention may be more prone to disruption in children.

## The Current Study

We addressed two main questions: First, though the bodies of literature examining the effects of salient visual events and memory on attention orienting have remained separate, people frequently encounter salient visual stimuli within familiar environments. When salient visual events occur in known environments, do children and adults shift their attention more in response to what they have learned from experience, or in response to novel events? Second, younger and older observers might differ in what guides their attention: Does the relative strength of each source of orienting change from childhood to adulthood, as memory, attention‐orienting, and cognitive control mechanisms continue to mature? To answer these questions, we adapted a memory‐guided orienting task (e.g., Summerfield et al., 2006) for use with a group of 7‐ to 12‐year‐old children and a group of healthy young adults. We chose this broad age range because there is yet no benchmark from other child studies, nor an a priori sense for how young floor effects or noncompliance might emerge. We therefore started with children as young as seven, as previous experience with comparably long attentional laboratory tasks (e.g., Shimi, Nobre, Astle, & Scerif, 2014; Shimi & Scerif, 2017) suggested children as young as 7 should be able to comply without being distressed or fatigued. Our upper limit of 12 was motivated by casting our net wide over childhood, in this first attempt to study children's memory‐guided orienting.

Participants first learned to associate naturalistic scenes with specific target locations by searching for small stars hidden within them. They then completed a test of explicit memory for the star locations. Finally, children and adults completed an attention‐orienting task in which they had to discriminate a small target. This target was preceded by both a scene with an associated memory, and a salient visual event (a flashing red box). The memory and visual event each cued the target on half the trials; critically, the validity of each cue type was independent so that we could examine whether the relative influence of each source of orienting differed across children and adults.

We expected children and adults to demonstrate evidence of both memory‐guided and exogenously cued attention. However, we expected that in the presence of salient visual events, given their more mature cognitive control networks (Johnson et al., 2014), adults would weigh past experiences more heavily than children in shaping their attentional deployment.

## Experiment 1

### Method

#### Participants

This study was approved by the Central University Research Ethics Committee at the University of Oxford. Thirty adults (*M*_age_ = 23.2 years, standard deviation (STD) = 3.3 years; range = 18.7–31.5 years; 24 females) and 27 children (*M*_age_ = 9.2 years, STD = 1.5 years; range = 7.0–12.9 years; 10 females), with normal or corrected‐to‐normal vision and no known history of psychiatric or learning disorders, participated in the study. An additional child was tested but excluded from analyses due to failure to complete all the tasks. Adult participants were recruited from the University of Oxford's undergraduate subject pool and from the surrounding community. They gave informed consent prior to participation in the study and were compensated at a rate of either £8 per hour or with research participation credit. Children were recruited via e‐mail advertisements sent to local schools in Oxfordshire, a primarily (> 90%) White‐British, middle‐class county. They gave informed assent and parents gave informed consent prior to their participation, and they received a certificate at the end of the testing session.

#### Stimuli

Stimuli for the task comprised 154 indoor and outdoor natural scenes acquired from Google images (1,920 × 1,080 pixels). The learning target was a small gold star prepared in Adobe Photoshop (15 × 15 pixels for adults; 25 × 25 pixels for children). The orienting targets included a small gray square (50 × 50 pixels) and circle (with a 50‐pixel diameter) prepared in Adobe Photoshop. The visual event at orienting comprised a 200 × 200‐pixel square, red outline.

#### Procedure

##### Learning phase

Adult participants sat 100 cm away from a 60 × 34‐cm monitor. On each trial, participants saw a new scene with an embedded target star. They were directed to search through each of the 88 scenes to find the star, clicking once they found it. After they clicked, a cursor would appear, so that participants could click on the star. Additionally, they were instructed to memorize the location of the stars that they found. Participants had 60 s to look through each scene, and 5s to move the mouse to click on the star. If participants successfully clicked within 50 pixels of the star, they then saw a feedback screen that said “Target Found” for 500 ms. If the scene timed out or if participants clicked on an incorrect location, they saw a feedback screen that said “Target Not Found” for 500 ms. Participants were given short breaks every 22 scenes. Participants completed three blocks of this procedure, with the same scenes in each block. At the end of each block, participants saw a screen that told them how many targets they found, and they had another opportunity to take a break. Children completed the same task, except that it was framed as a game featuring the Nintendo character Mario. Additionally, to decrease experiment duration, the stars were made easier to locate by increasing their size (from 15 × 15 pixels to 25 × 25 pixels). Children were told that Mario had lost his stars and needed help. When children located the star and clicked the mouse, a small image of Mario appeared, serving as the cursor. Children could move Mario to collect the star by moving the mouse and clicking. Prior to beginning the task, children completed one trial in which they practiced this entire procedure.

##### Location memory test

After a short break (3–5 min), participants were tested on their explicit memory for the target locations. Participants viewed the 88 scenes from the learning phase in a random order, but this time the stars were absent from the scene. Participants were instructed to click when they remembered where the star was, and then to move the cursor to the remembered location and click again. They had 30 s to initiate the first mouse click, and an additional 10 s to move the cursor. Children completed the same test of explicit memory, except they were told that they were responsible for informing Mario's friend, Luigi, of the star locations. When children clicked the mouse, Luigi appeared, and they had to move him over to the location of the star and click again. Children completed one practice trial prior to beginning the real task.

##### Orienting

###### Memory and visual event interactions

**Figure 1:**
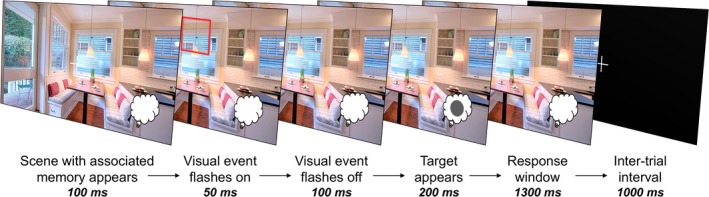
Orienting task: memories and visual events. In the first orienting block, participants saw a scene with an associated memory for 100 ms. Then, a red square appeared for 50 ms. After a brief delay (100 ms), a target square or circle appeared, which participants were instructed to discriminate. [Color figure can be viewed at wileyonlinelibrary.com]

After the location memory test, participants completed the first block of the orienting task. Throughout the task, participants were told to respond to a small gray square or circle target that appeared on every trial using a standard keyboard as quickly and as accurately as possible by pressing either the up key for squares and the down key for circles. Square and circle stickers were taped over the appropriate keys. For the first 100 ms of every trial, participants viewed a scene with an associated memory from the learning phase. Then, a transient, visual event, which comprised a red box appeared within the scene (50‐ms duration). After the visual event disappeared, the scene remained on the screen for another 100 ms, after which the gray target appeared briefly within the scene (200 ms duration). The scene then remained on the screen for an additional 1,300 ms. Participants could respond to the target any time between its onset and the end of this 1,300 ms window (Figure 1).

In summary, the memory cue (scene) onset preceded target onset by 250 ms, whereas the visual events preceded target onset by 150 ms. These timings were chosen based on previous experiments in order to optimize effects of both cue types (Brodeur & Enns, 1997; Eimer, 2000; Summerfield et al., 2006).

In the first orienting block, cues were orthogonalized such that the target appeared in either a location cued by both the memory (i.e., where the star was previously) and the visual event (*MV*), cued by the memory only (*M*), cued by the visual event only (*V*), or in a fully un‐cued location (*U*). Un‐cued locations were randomly selected locations on the opposite hemifield from the visual or memory cues. All trial types were intermixed, and the order of the trials was randomized. Participants had the chance to take short breaks every 22 trials. Participants were told that both the memory and the visual event would sometimes, but not always, indicate the target location. They were also instructed to keep their eyes fixated on the central cross that appeared on top of all orienting stimuli. Before beginning the real trials, adult participants completed 10 trials to practice the button‐press responses in which they saw the gray targets appear on top of a black screen. Children completed the same task, but they were told that the gray squares and circles were stones that Mario needed to build his castle. After every set of 22 trials, they were shown a picture of a castle in various stages of completion, until the end, in which they saw the whole castle. Additionally, children completed 20 practice trials before beginning the task.

###### Visual events only

The second orienting block was identical to the first except that all scenes used were novel. This block therefore served the purpose of establishing whether there were any age‐related differences in visual event cuing effects, when cues and targets were embedded in novel, natural scenes. Participants completed 66 trials. One third of trials (22) were neutral (*VN*) such that no visual event was included prior to target onset. The target appeared on the scene after a 250 ms delay. The remaining two‐thirds of trials (44) included the same visual event as the first orienting block (the red box). On half of these trials (22), the target appeared in a location cued by the visual event, and on half of these trials (22), the target appeared in an un‐cued location on the opposite side of the screen. We will refer to these as visual‐only cued (*VC*) and visual‐only un‐cued (*VU*) trials. As in the first orienting block, participants were given breaks every 22 trials.

#### Counterbalancing

Presentation of the scenes was counterbalanced across participants, such that they appeared with equal chance in the four orienting conditions in the first orienting block (*MV*,* M*,* V*,* U*), and the three orienting conditions in the second orienting block (*VN*,* VC*,* VU*). The location of the learning target was counterbalanced across both participants and scenes, such that each participant saw half of the learning targets (44) on the right and half (44) on the left, and for each scene the target appeared on the right for half of the participants and the left for half of participants. Within each half of the screen, the location of the learning target was randomized. The location of the orienting target and the identity of the orienting target was randomized for each scene but counterbalanced within conditions for each participant.

#### Variables of Interest

##### Learning phase

Three variables were extracted as measures of participants’ learning of the target locations across the three blocks: missed‐targets *z*‐scores, search‐time *z*‐scores, and search‐time slope. Missed targets were the number of targets participants failed to find in each learning block. Search time was calculated as the time from scene onset to first mouse click. Only trials in which the target was found across all three blocks were included in the search‐time analysis (mean number included = 75.2 trials; range = 53–85 trials). Because children and adults viewed learning targets of slightly different sizes, rather than using raw scores, we *z*‐scored these variables to index the extent to which each participant improved relative to their own baseline throughout the learning phase. To index how performance improved over the course of the three blocks, we also computed slope values for search times for each participant.

##### Location memory test

Two measures were extracted to determine participants’ explicit memories for the target locations: memory precision and memory accuracy. Memory precision was calculated as the distance in pixels between where the participant clicked during the memory test and the true center of the target location during the learning phase. Trials in which participants failed to find the target at least once during the learning phase were excluded from the analysis (mean number included = 84.8 trials; range = 78–88 trials). Trials were considered “accurate” if participants clicked within an imaginary 200 × 200‐pixel box centered on top of the target location.

##### Orienting

The first and second orienting blocks were analyzed separately. For the first block, only trials in which the target was found at least once during the learning phase were included (mean number included = 84.8 trials; range = 78–88 trials). Three measures were extracted to determine the effects of cuing condition on attention orienting: accuracy, accuracy with missed trials excluded, and reaction time. Trials were considered accurate if participants made a correct response within the 1,500‐ms response window. Trials were considered inaccurate if participants failed to respond or if they pressed the wrong button. To calculate accuracy with missed trials excluded, we ignored all trials in which participants failed to respond. Even when missed trials were excluded, the performance of three children was still statistically indistinguishable from chance level (chance level: 50%). Thus, they may not have tried to perform the task correctly and instead pressed random buttons. As such, these participants were excluded from orienting analyses. Reaction time was calculated as the time from target onset to button‐press response. Only accurate trials were included (Block 1 [88 trials] mean number included = 64.39 trials; range = 45–78 trials; Block 2 [66 trials]: mean number included = 52.5 trials; range = 37–62 trials).

### Results

#### Successful Learning of Target Locations

Converging evidence from our targets‐found and search‐time analyses suggested that both children and adults learned the location of the targets across search blocks. A repeated‐measures analysis of variance (ANOVA; with Greenhouse–Geisser correction, applied as necessary in subsequent analyses) examining the effects of learning block and age group on search‐time *z*‐scores revealed that both children and adults demonstrated faster search times across learning blocks, *F*(1.13, 62.41) = 805.85, *p *<* *.001, $\eta_{p}^{2}$ = .936. There was No Learning Block × Age Group interaction, *F*(1.13, 62.41) = 2.08, *p* = .13, $\eta_{p}^{2}$ = .036. Additionally, a repeated‐measures ANOVA examining *z*‐scores for the number of targets missed per learning block as a within‐subjects factor and age group as a between‐subjects factor revealed that both children and adults found increasing numbers of targets as the task proceeded, *F*(2, 110) = 51.78, *p *<* *.001, $\eta_{p}^{2}$ = .485. There was No Learning Block × Age Group interaction, *F*(2, 110) = 0.86, *p* = .201, $\eta_{p}^{2}$ = .029, indicating that children and adults demonstrated comparable improvement in finding targets across blocks.

#### Explicit Memories for Target Locations

We next analyzed whether children and adults formed explicit memories for the target locations. There was not a significant difference in memory accuracy between adults (*M* = .67, *SD* = .17) and children (*M* = .58, *SD* = .20), *t*(51.4) = 1.7, *p* = .068. However, independent‐samples *t*‐tests indicated that adults had more precise memories for the target locations (*M* = 145.1 pixels, *SD* = 79.1 pixels) than children (*M* = 203.5 pixels, *SD* = 126.9 pixels), *t*(42.68) = 2.1, *p* = .045. Additionally, we examined whether individual participants’ search‐time slopes correlated with their memory precision on the explicit memory test. These two measures were negatively correlated in both adults, *r*(28) = −.60, *p *<* *.001, and children, *r*(25) = −.48, *p* = .012, indicating that participants who showed greatest evidence of learning (*greater* slope values) during the first part of the experiment also demonstrated the greatest memory precision (*lower* values indicate higher precision) in the second part. These data suggest that the increasing search speeds we observed across learning blocks index learning of the target locations.

#### Effects of Visual Events on Attention Orienting

We next wanted to ensure that our visual events were salient enough to engage exogenous attention‐orienting mechanisms when they appeared on top of scene stimuli. To address this question, we analyzed the data from our second orienting block, in which a salient, visual event occurred as a novel scene was presented. To account for subject‐level differences in orienting effects, we analyzed participants’ orienting accuracy with logistic mixed‐effects models and their reaction times with linear mixed‐effects models using the Analysis of Factorial Experiments (afex) package (Version 0.19‐1) in R (Version 3.4.2; R Core Team, 2014; Singmann, Bolker, & Westfall, 2015). This approach enabled us to include all trials for each participant, rather than just the means of aggregated data (e.g., Baayen, Davidson, & Bates, 2008). To determine the random‐effects structure for our models that would both minimize Type I error and maximize power, we followed the “forward‐stepping‐best‐path” approach recommended in Barr, Levy, Scheepers, and Tily (2013; Barr et al., 2013). Our final accuracy model included our fixed effects of interest (visual event condition with three levels: neutral (*VN*), cued (*VC*), un‐cued (*VU*); and age group with two levels: adults, children), random intercepts for subject and scene, and allowed for different slopes across visual event conditions for each scene. A likelihood ratio test indicated that including the visual event condition significantly improved model fit, χ^2^ = 66.97, *p *<* *.0001. To determine whether these accuracy effects were driven by a performance advantage in the cued (*VC*) condition versus a performance impairment in the un‐cued (*VU*) condition, we compared accuracy across conditions using Tukey contrasts. Results indicated that participants were more accurate in the cued and neutral conditions relative to the un‐cued condition, *p*s < .0001. There was no reliable difference in accuracy between the cued and neutral conditions, *p* = .711. Additionally, including age group also improved model fit, χ^2^ = 13.63, *p* = .0002. Across conditions, children performed less accurately than adults (Table 1). There was no effect of including the Visual Event Condition × Age Group interaction term in the model, χ^2^ = 1.13, *p* = .57, indicating that both children and adults demonstrated the same pattern of attentional costs on un‐cued trials.

**Table 1:** Experiment 1: Means (and Standard Deviations) of Accuracy and Reaction Times as a Function of Age Group and Validity of Exogenous Cue for the Visual‐Cue‐Only Orienting Block

Age group	Visual cue	Accuracy	Reaction time (ms)
Adults	Neutral	.86 (.35)	619.2 (168.6)
	Cued	.88 (.33)	597.5 (166.7)
	Un‐cued	.76 (.43)	660.3 (210.2)
Children	Neutral	.78 (.42)	832.7 (202.8)
	Cued	.83 (.38)	816.4 (215.7)
	Un‐cued	.63 (.48)	866.5 (246.4)

**Figure 2:**
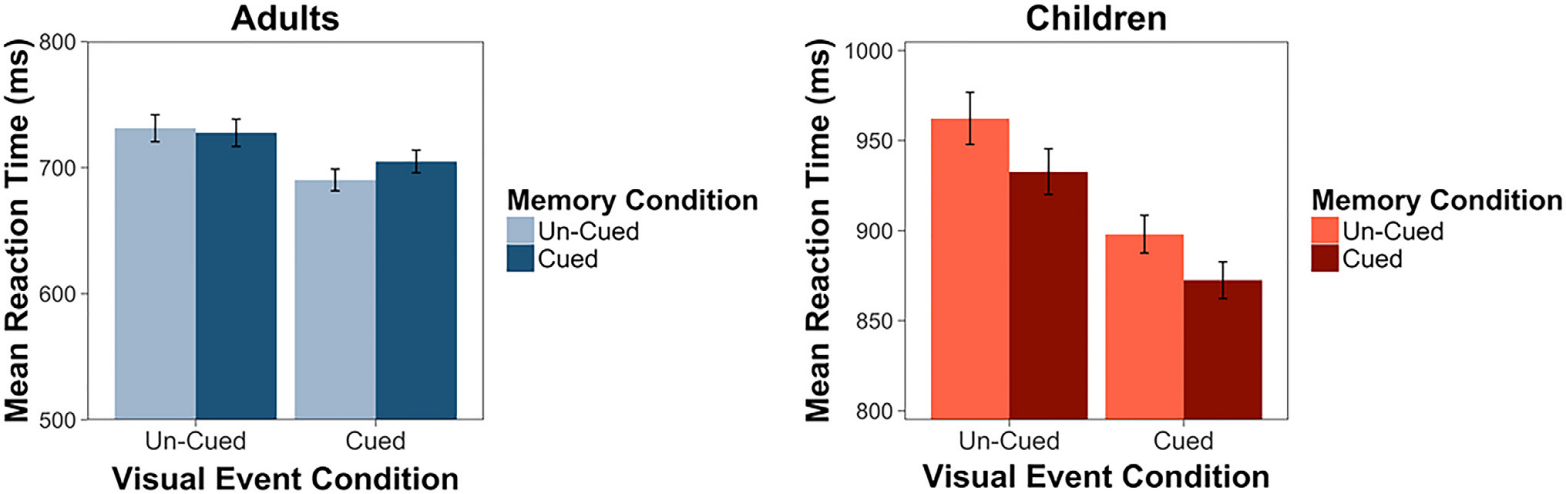
Experiment 1. Adults and children both demonstrated faster reaction times when the visual event cued the target location, p < .0001. However, only children demonstrated similar cuing effects in response to memories, demonstrating faster reaction times when the memory cued the target location, p = .007. Error bars indicate standard errors.

We followed the same process to analyze our reaction‐time data, but we tested the significance of our effects using *F* tests with Kenward–Roger approximations for degrees of freedom. Our final model included random intercepts for subject and scene and allowed for different slopes across visual event conditions for each subject and each scene. Similar to our accuracy data, we observed a main effect of visual event condition on orienting reaction times, *F*(2, 61.23) = 9.91, *p* = .0002. Tukey contrasts revealed that participants performed slower on un‐cued (*VU*) trials relative to both cued (*VC*) trials (*p* = .0001) and neutral (*VN*) trials (*p* = .02; Figure 2). There was no difference in reaction times across cued and neutral trials, *p* = .133. As with our accuracy analysis, there was also a main effect of age group on reaction times, *F*(1, 49.86) = 54.71, *p *<* *.0001, with children demonstrating overall slower reaction times than adults (Table 1). The Cue Condition × Age Group interaction effect was not significant, *F*(2, 47.96) = 0.23, *p* = .79. These results indicate that both children and adults showed a comparable effect of visual events on orienting performance, with the onset of a visual event impairing performance when it did not cue the target location.

#### Interactions Between Memory Cues and Visual Events on Attention Orienting

Finally, after verifying that children and adults (a) learned to associate target locations with specific scenes, and (b) demonstrated differences in orienting accuracy and reaction time depending on whether the visual event cued the target location, we examined our results from the first orienting block to address our main question of interest: how memories and visual events *interact* to influence attention orienting. As before, we examined participants’ orienting accuracy and reaction times with mixed‐effects models. Both models included memory condition, visual‐event condition, and age group as fixed effects, subject and scene as random intercepts, and allowed for different slopes across visual event conditions for each subject. Our accuracy model additionally allowed for different slopes across memory conditions for each scene, and our reaction time model allowed for different slopes across visual event conditions for each scene.

As indicated in Table 2, the only reliable predictors of orienting accuracy were visual‐event condition and age. Visual events that cued the target location led to increased accuracy, and adults performed better than children. We observed no effects of memory condition on orienting accuracy, nor a Memory Condition × Age Group interaction.

**Table 2:** Experiment 1: Orienting Accuracy Mixed Effects Model: Coefficients and Chi‐Square Values

Effect	Coefficient (*SE*)	χ^2^	*p*
Intercept	1.34 (.08)		
Visual‐event condition***	−0.55 (.06)	54.21	< .0001
Memory condition	−0.06 (.05)	1.33	.25
Age group**	0.25 (.07)	10.95	.0009
Visual‐Event × Memory Condition	0.00 (.04)	0.00	.98
Visual‐Event Condition × Age Group	0.04 (.06)	0.63	.43
Memory Condition × Age Group	0.00 (.04)	0.00	.98
Visual‐Event Condition × Memory Condition × Age Group	0.01 (.04)	0.06	.81

**p < .01. ***p < .001.

As with our accuracy model, our reaction‐time model indicated that the visual‐event condition was a reliable predictor of orienting reaction times (Table 3), with participants responding more quickly on trials in which the visual event cued the target location. Here though, we also observed a significant effect of memory condition on participant reaction times, which was qualified by a significant Memory Cue Validity × Age Group interaction effect (Table 3).

**Table 3:** Experiment 1: Orienting Reaction Time Mixed‐Effects Model: *F* Test With Kenward–Rogers Approximations for Degrees of Freedom

Effect	*df*	*F*	*p*
Visual‐event condition***	1, 52.57	26.23	< .0001
Memory condition*	1, 3,279.23	3.98	.05
Age group***	1, 51.98	37.34	< .0001
Visual‐Event Condition × Memory Condition	1, 3,275.55	0.58	.45
Visual‐Event Condition × Age Group	1, 51.72	0.63	.43
Memory Condition × Age Group**	1, 3,207.38	6.63	.01
Visual‐Event Condition × Memory Condition × Age Group	1, 3,203.73	0.22	.64

*p < .05. **p < .01. ***p < .001.

We further investigated this interaction with separate models for each age group. Contrary to our initial hypothesis, children, but not adults, responded more quickly to targets cued by memories, *F*(1, 1,392.72) = 7.33, *p* = .007. Adults did not demonstrate any effects of memory cue condition on orienting reaction times, *F*(1, 1,869.23) = 0.12, *p *=* *.73 (Figure 2). These analyses indicate that when visual events occurred within familiar contexts, children's attention orienting was influenced by *both* sources, whereas adults were influenced by the visual events only, *despite* adults demonstrating greater precision in their memories for target locations.

### Experiment 1 Discussion

The results of Experiment 1 demonstrated that, although both children and adults learned to associate specific target locations with scenes, only children used these memories to orient their attention when novel visual events also preceded the onset of visual targets. These data stand in stark contrast to previous findings from the contextual cuing literature (e.g., Couperus et al., 2011; Vaidya et al., 2007), which point to stronger effects of memory on attention in adults, particularly in the presence of potential interference.

Given that many past studies using similar task designs have found that adults *do* demonstrate memory‐guided attention orienting (Patai et al., 2012; Salvato et al., 2016; Stokes et al., 2012; Summerfield et al., 2006), our results suggest that the salient, visual events may have *disrupted* or *overwritten* the effects of memory‐guided attention in adults but not in children. Interestingly, however, the results from our visual‐events‐only orienting block indicated that adults and children demonstrated equivalent costs on un‐cued trials across both accuracy and reaction time measures. Thus the differences that we observed in the interaction between memory‐guided and exogenously cued attention across adults and children cannot be attributed to differences in their sensitivity to the visual events on their own.

There are several possible explanations for why children used memories to orient their attention in the face of competing visual cues but adults did not. First, it is possible that the design specifications of our task were not sensitive enough to elicit or reveal memory‐guided attention in adults. Though we based our experimental design on published studies that have shown memory‐guided attention in adults, it is nevertheless important to verify that our task was sensitive enough to elicit memory‐guided attention in adults in the absence of visual events. Additionally, it is also possible that adults may have strategically ignored the memory cues. In our task, memories and visual events each correctly indicated the target location on half of trials. Since there were more than two possible locations at which the target could appear, paying attention to the cues would be an adaptive strategy. However, over the course of the orienting task, dividing attention over multiple cued locations would not have accrued participants a much larger benefit than if they strategically ignored one cue type entirely. Past work suggests that adults are better than children at strategically modifying their responses to visual cues depending on their predictive validity (Brodeur & Boden, 2000; Enns & Brodeur, 1989; Iarocci et al., 2009), so it is possible then that adults strategically ignored the memory cues while children did not.

To probe these two possibilities, we conducted a second experiment in adults. We sought to: (a) assess whether the presence of visual events interfered with memory‐guided attention in adults, and (b) determine if the presence of visual events prompted adults to strategically ignore the memory cues. To test these possibilities, we included orienting trials in which only scenes with associated memories appeared, as well as orienting trials in which the visual events *never* indicated the location of the subsequent target. If the presence of visual events had disrupted memory‐guided attention in adults, we expected that we would see effects of memories on attention orienting when visual events were not present. If the visual events disrupted memory‐guided orienting by prompting adults to ignore strategically the memories in favor of the visual events, we expected to mitigate this disruption by replacing the visual events with nonpredictive distractors.

## Experiment 2

### Method

#### Participants

Twenty‐seven adults (*M*_age_ = 23 years, STD = 2.6 years; range = 19.4–28.8 years; 19 females) with normal or corrected‐to‐normal vision and no known history of psychiatric or learning disorders participated in the study. One additional adult was tested but excluded from all analyses due to clicking through the scenes without searching in the learning phase. Participants were recruited, consented, and compensated as in Experiment 1.

#### Stimuli

Stimuli for the task were the same as those used in Experiment 1.

#### Procedure

##### Learning phase and location memory test

Participants completed the same learning phase and location memory test as in Experiment 1.

##### Orienting

The orienting task was the same as that in the first experiment with three key differences. First, in the first *memories‐only* block (44 trials), no visual events were presented. Second, in the *memory cue and visual distractor* block (44 trials), each trial included a scene with an associated memory as well as a visual distractor. The memory cued the target location on half of trials (22). The visual distractors *never* indicated the subsequent target location, and participants were explicitly instructed to ignore them. The distractor locations were randomized on each trial with the constraint that within each memory condition, half of the trials included a *near* distractor that appeared in the same hemifield as the target and half of the trials included a *far* distractor that appeared in the opposite hemifield as the target. This constraint was placed to ensure that the average distance between the target and the distractor was similar across memory conditions. Third, in the *visual‐distractor only* block (66 trials), distractors were presented on top of novel scenes. Thirty‐three trials included no distractor (neutral trials), and 16 or 17 trials included a near distractor, and the remaining 16 or 17 trials included a far distractor. As in the second orienting block, participants were explicitly instructed to try to ignore the distractor.

#### Counterbalancing

As in Experiment 1, the location of the learning target and the orienting condition of the scenes and targets was counterbalanced across participants. Within conditions with distractors, whether scenes appeared with a near or far distractor was randomized.

#### Variables of Interest

We extracted variables from each phase of the experiment as in Experiment 1. For the learning phase, since we were no longer comparing two groups that had completed slightly different versions of the task, we used raw data rather than *z*‐scores.

### Results

#### Successful Learning of Target Locations

As in Experiment 1, repeated‐measures analyses of variance examining the effects of learning block on targets missed and search times indicated that participants found targets increasingly accurately, *F*(1.64, 42.55) = 39.26, *p *<* *.001, $\eta_{p}^{2}$ = .602, and quickly, *F*(1.46, 37.89) = 209.95, *p *<* *.001, $\eta_{p}^{2}$ = .890, over the course of the learning phase.

#### Explicit Memory for Target Locations

Participants correctly remembered an average of 70.2% (*SD* = 13.6%) of the target locations with an average precision of 131.4 pixels (*SD* = 61.9 pixels). As in Experiment 1, participants’ search‐time slopes correlated with their memory precision, *r*(25) = −.40, *p* = .038, such that participants whose speed improved the most over the course of the three blocks also demonstrated more precise memories for the target locations.

#### Effects of Memories on Attention Orienting

**Figure 3:**
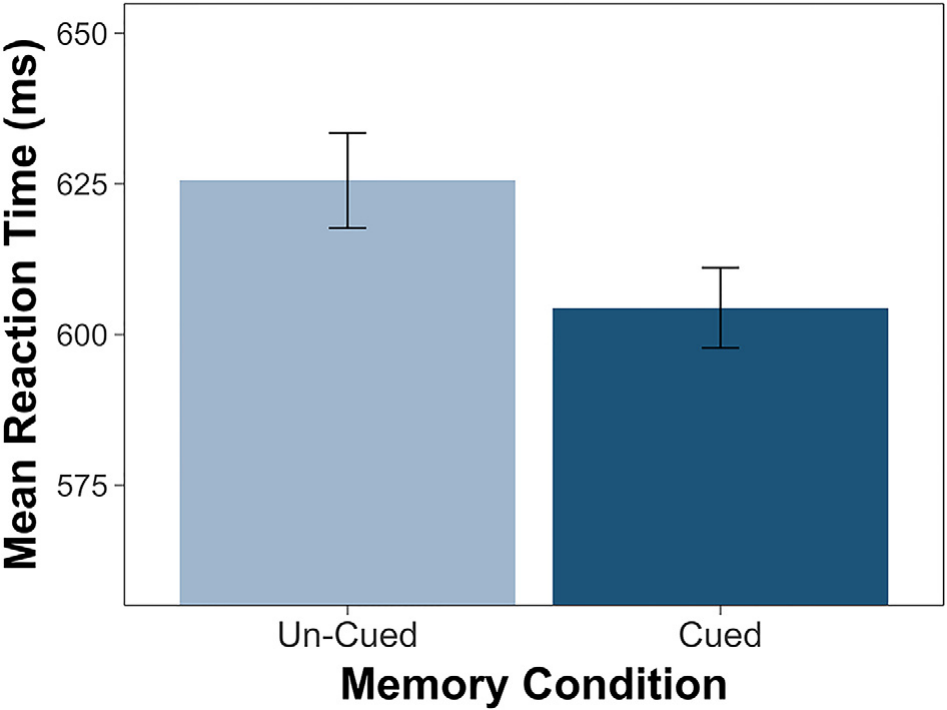
Experiment 2. In the absence of the sudden onset of novel visual events, adults demonstrated faster orienting reaction times to targets presented in locations cued by memories, relative to un‐cued locations (p = .03). Error bars indicate standard error. [Color figure can be viewed at wileyonlinelibrary.com]

After verifying that participants were able to learn and remember locations associated with scenes, we examined whether these memories influenced attention orienting in the absence of additional visual events. As in Experiment 1, we constructed a logistic mixed‐effects model to examine the effects of memory condition (cued, un‐cued) on orienting accuracy, and a linear mixed‐effects model to examine the effects of cue condition on orienting reaction time. All our orienting models included memory condition (cued, un‐cued) and, when relevant, distractor location (near, far) as fixed effects and subject and scene as random intercepts. Our first two models further included different slopes across memory conditions for each scene. A likelihood‐ratio test revealed no effect of memory cuing on orienting accuracy, χ^2^(1) = 0.82, *p* = .37. However, participants’ responses were faster when target locations were cued by memories, *F*(1, 142.91) = 4.27 *p* = .04 (Figure 3). Thus, without the onset of novel visual events, we observed the expected benefits of memory cuing on attention orienting in adults.

#### Effects of Distractors on Attention Orienting

Next, we examined the results from our third orienting block, to determine how visual distractors influenced attention orienting. Results revealed an effect of distractor location on orienting accuracy, χ^2^(2) = 15.70, *p* = .0004, with participants responding most accurately when no distractor was present and least accurately when a far distractor appeared (Table 4). Post hoc Tukey contrasts revealed that participants responded less accurately on far‐distractor trials relative to no‐distractor trials (*p *<* *.001). There was no difference in participants’ accuracy across no‐distractor and near‐distractor trials (*p* = .346), or across near‐distractor and far‐distractor trials (*p* = .083). We also observed an effect of distractor location on orienting reaction times, *F*(2, 25.76) = 6.54, *p* = .005. Post hoc Tukey contrasts revealed that responses were significantly faster on no‐distractor trials, relative to trials with either near or far distractors (*p*s < .04). There was no difference in reaction times across near‐ and far‐distractor trials (*p* = .955). Taken together, these results indicate that our visual distractors operated much like the visual events on un‐cued trials in Experiment 1: They impaired orienting accuracy and speed, even when they did not provide any useful predictive information and participants were explicitly instructed to ignore them.

**Table 4:** Experiment 2: Means (and Standard Deviations) of Accuracy and Reaction Times as a Function of Orienting Condition Across Experiment Blocks

Block	Memory condition	Visual distractor location	Accuracy	Reaction time (ms)
Memories only	Cued	N/A	.87 (.34)	604.4 (144.9)
	Un‐cued		.89 (.32)	625.6 (174.5)
Visual distractors only	N/A	No distractor	.90 (.31)	598.8 (168.8)
		Near	.86 (.35)	657.9 (178.9)
		Far	.79 (.41)	643.2 (191.6)
Memories and visual distractors	Cued	Near	.85 (.36)	666.1 (179.0)
		Far	.76 (.43)	661.5 (195.6)
	Un‐cued	Near	.87 (.33)	668.3 (188.7)
		Far	.79 (.41)	663.8 (198.0)

#### Interaction Between Memory Cues and Distractors on Attention Orienting

Finally, we sought to determine whether the addition of distractors would disrupt memory‐guided attention‐orienting in adults, or whether their failure to use memory cues in Experiment 1 was due to adults' strategically ignoring them in favor of the predictive, visual events. There was a significant effect of distractor location on orienting accuracy, χ^2^(1) = 17.92, *p *<* *.0001, with participants responding more accurately when distractors were presented near the target location, as they did in the block when no memories were present (Table 4). There was no effect of memory condition on orienting accuracy, χ^2^(1) = 0.87, *p* = .35, nor was there a Memory Condition × Distractor Location interaction effect, χ^2^(1) = 0.09, *p* = .76. Memory condition similarly did not influence orienting reaction times, *F*(1, 136.62) = 0.02, *p* = .88. Additionally, there was no effect of distractor location on orienting reaction times, *F*(1, 244.90) = 0.06, *p* = .80, nor was there a Memory Condition × Distractor Location interaction effect, *F*(1, 261.37) = 0.00, *p* = .99. Taken together, these results indicate that, like the visual events Experiment 1, the onset of visual distractors disrupted memory‐guided attention in adults.

### Experiment 2 Discussion

In Experiment 2, we observed intact memory‐guided attention in adults when no visual distractors were present, as evidenced by faster reaction times to targets presented in memory‐cued relative to memory‐un‐cued locations. However, the addition of a nonpredictive, visual distractor disrupted this effect, such that we observed no influence of memory condition on orienting accuracy or reaction times, even when participants were explicitly instructed to ignore the distractor and only orient attention based on the memory. Additionally, when presented alone, both near and far distractors significantly impaired orienting speed, indicating that attending to them would not have been an adaptive strategy.

These results help clarify the interpretation of Experiment 1 in two important ways. First, they indicate that, in the absence of visual events, adults did indeed demonstrate memory‐guided attention. This suggests that the parameters of our task were sensitive enough to reveal memory‐guided orienting. Second, the fact that adults failed to demonstrate any influence of memory on attention in the presence of nonpredictive visual events—even as they were explicitly instructed to ignore these distractors—suggests that the developmental differences in attention that we observed in Experiment 1 were unlikely to be due to differences in strategy use across children and adults.

At first blush, it seems surprising that competent attentive observers such as young adults should continue to be distracted by sudden visual events, even when they know that these events are not predictive and they are explicitly asked to ignore them. However, previous work has shown that exogenous events are difficult to ignore, even when they do not predict the location of stimuli that should be attended (Berger, Henik, & Rafal, 2005; Theeuwes & Godljn, 2002). Given this previous work, we did not expect adults to be able to suppress fully the distractors, but we did expect that they would be more likely to use the memory cues if they knew they were the only source of predictive information in the task. Our data from the first orienting block, in which only memory cues were present, support this prediction: Adults did use memories to orient attention when they correctly predicted the target location on 50% of trials. Thus our results suggest that adults’ failure to use memories to guide attention in Experiment 1 was *not* due to them strategically modulating their recall or use of the memory associations.

Though Experiment 2 largely ruled out differences in strategy use as an explanation for why children but not adults are able to use memories to guide attention in the presence of transient visual events, it still leaves open the question of what accounts for the developmental differences we observed in Experiment 1. One possibility is that there may be baseline differences in the extent to which memories influence attention *in the absence of transient visual events* across children and adults. It could be the case that, contrary to predictions, children show *larger* memory‐guided orienting effects, such that they remain detectable even when dampened by interfering, visual events. Previous literature has described memory‐guided attention in children as less robust than memory‐guided attention in adults (Couperus et al., 2011; Vaidya et al., 2007). In large part, this description has arisen from the mixed findings for memory‐guided attention in children across different studies (Couperus et al., 2011; Dixon et al., 2010; Vaidya et al., 2007; Yang & Merrill, 2015). However, very few studies have directly compared memory‐guided attention in children and adults using the same tasks across age groups. One (Merrill et al., 2013) found that the magnitude of contextual cuing effects was equivalent across young 6‐ to 7‐year‐old children and young adults, whereas other studies have demonstrated that children fail to show contextual cuing effects on paradigms in which adults do (Couperus et al., 2011; Darby et al., 2014; Vaidya et al., 2007). Dixon et al. (2010), however, found a trend toward larger memory benefits for younger children. In the auditory domain, one study did find that when both children and adults were instructed to respond to a specific tone, children demonstrated *greater* reaction time benefits than adults when tones were cued by implicitly learned sequences (Ruhnau, Schröger, & Sussman, 2017). In the visuospatial domain, to the best of our knowledge, no work has found that children demonstrate greater memory‐cuing effects relative to adults. Furthermore, as previously mentioned, all published work on memory‐guided attention in children has used contextual cuing paradigms. As such, it is impossible to determine whether any developmental differences observed in these tasks are due to differences in learning rate, memory fidelity, or the *use* of memories to guide attention.

To address these questions, we conducted an experiment in which a new group of school‐aged children and new a group of healthy, young adults completed identical tasks, modeled after those used in Experiment 1. Here, adults also completed the “gamified” version of the tasks with Mario characters, to mitigate any differences in performance that may have arisen from the child stimuli being more rewarding (Doallo, Patai, & Nobre, 2013). Furthermore, adults and children searched for targets of the same size during the learning phase, so that learning rates and explicit memory could be compared across age groups in the absence of any potentially confounding effects of task differences. Finally, in this version of the task, no transient visual events occurred at orienting. Instead, all participants simply saw a scene with an associated memory for 250 ms prior to target onset. We hypothesized that participants in both age groups would demonstrate an influence of memory on attention orienting, but that in the absence of salient visual events, the size of this memory cueing benefit would be greater in adults relative to children, in line with past literature that has demonstrated more consistent effects of memories on attention in adults relative to children.

## Experiment 3

### Method

#### Participants

Twenty‐eight adults (*M*_age_ = 20.0 years, STD = 1.4 years; range = 18.4–25.2 years; 17 females) with normal or corrected‐to‐normal vision and no known history of psychiatric or learning disorders, participated in the study. Twenty‐seven school‐aged children (*M*_age_ = 9.0 years, STD = 1.4 years; range = 7.1–11.7 years; 13 females) also participated. Six additional children were tested but excluded from all analyses due to failure to complete all the tasks. One child's data were excluded from the orienting analyses due to failure to respond on 75% of trials.

#### Stimuli

Stimuli for the task comprised a subset of those used in Experiments 1 and 2. This experiment comprised only one orienting block with 88 trials. As such, we selected 88 scene images from the group of stimuli used in the prior experiments.

#### Procedure

##### Learning phase and location memory test

Participants completed the same learning phase and location memory test as the children completed in Experiment 1. Stars were 15 × 15 pixels for both children and adults.

##### Orienting

As in our previous experiments, participants were told to discriminate a small, gray square or circle target that appeared on every trial using a standard keyboard. On every trial, participants first viewed a scene with an associated memory. Then, after a 250‐ms delay period, the target appeared for 200 ms. Participants had an additional 1,300 ms to respond to the target, during which time the scene remained on the screen. On half (44) of the 88 orienting trials, the scene with the associated memory cued the target location. On the other half (44) of trials, the target appeared at a random location on the opposite hemifield of the screen as the location cued by the memory. Presentation of the scene conditions, target locations, and target identities were counterbalanced across participants as in previous experiments.

#### Variables of Interest

##### Learning phase and location‐memory test

When we compared learning performance across children and adults in Experiment 1 we used *z*‐scores; however, here children and adults searched for targets of an identical size. In order to examine absolute differences in search speeds across age groups, we did not normalize our variables, and instead used the raw scores. All other variables examined were the same as those in Experiment 1.

### Results

#### Successful Learning of Target Locations

Converging evidence from our targets missed and search‐time analyses suggest that both children and adults learned the location of the targets across search blocks. A repeated‐measures ANOVA examining the number of targets missed with learning block as a within‐subjects factor and age group as a between‐subjects factor revealed that both children and adults found increasing numbers of targets as the task proceeded, *F*(1.8, 94.3) = 163.0, *p *<* *.0001, $\eta_{p}^{2}$ = .755. There was also a main effect of age group, with children missing more targets than adults across blocks, *F*(1, 53) = 26.67, *p *<* *.0001, $\eta_{p}^{2}$ = .335. Finally, there was also a Block × Age Group interaction, *F*(1.8, 94.3) = 7.43, *p* = .001, $\eta_{p}^{2}$ = .123, with children demonstrating greater improvement across blocks relative to adults. A repeated‐measures ANOVA examining the effects of learning block and age group on search times revealed that both children and adults demonstrated faster search times as learning progressed across blocks, *F*(1.5, 81.2) = 283.77, *p *<* *.0001, $\eta_{p}^{2}$ = .843. Additionally, adults demonstrated overall faster search times across blocks, *F*(1, 53) = 15.0, *p* = .0003, $\eta_{p}^{2}$ = .221. Here, however, there was no Learning Block × Age Group interaction, *F*(1.5, 81.2) = 2.9, *p* = .075, $\eta_{p}^{2}$ = .051.

#### Explicit Memories for Target Locations

We next analyzed whether children and adults formed explicit memories for the target locations to a similar extent. One‐sample *t*‐tests examining memory accuracy revealed that accuracy for both groups was significantly above chance level (4%); adults: *t*(27) = 17.47, *p *<* *.0001; children: *t*(26) = 15.95, *p *<* *.0001. There was no difference in memory accuracy between adults (*M* = .72, *SD* = .45) and children (*M* = .66, *SD* = .48), *t*(53.0) = 1.06, *p* = .29, nor was there a difference in memory precision between adults (*M* = 132.9 pixels, *SD* = 224.4 pixels) and children (*M* = 171.2 pixels, *SD* = 269.8 pixels), *t*(51.7) = 1.21, *p* = .23. These data suggest that adults and children formed comparably precise memories for the target locations. Additionally, we examined whether participants’ search‐times slopes—measures of their ability to learn the location of the targets throughout the learning phase—correlated with their memory precision on the explicit location memory test. For both adults and children, there were significant correlations between participants’ search‐time slopes and memory precision; *adults: r*(26) = −.38, *p* = .044, *children: r*(25) = −.87, *p *<* *.0001.

#### Effects of Memories on Attention Orienting

We next turned to our main question of interest: How do memories influence attention across children and adults? To address this question, we analyzed orienting accuracy and reaction times across memory cue conditions and age groups. To determine how memory condition influenced orienting accuracy across age groups we constructed a logistic mixed‐effects model. Our final model included our fixed effects of interest (memory condition with two levels: cued and un‐cued; age group with two levels: adults and children), random intercepts for subject and scene, and allowed for different slopes for age groups across scenes. A likelihood‐ratio test indicated that including age group improved model fit, χ^2^(1) = 37.6, *p *<* *.0001. Across conditions, children (*M* = .72, *SD* = .45) responded less accurately than adults (*M* = .86, *SD* = .34). There was no effect of memory condition on orienting accuracy, nor was there an Age Group × Memory Condition interaction effect, *p*s > .25.

**Figure 4:**
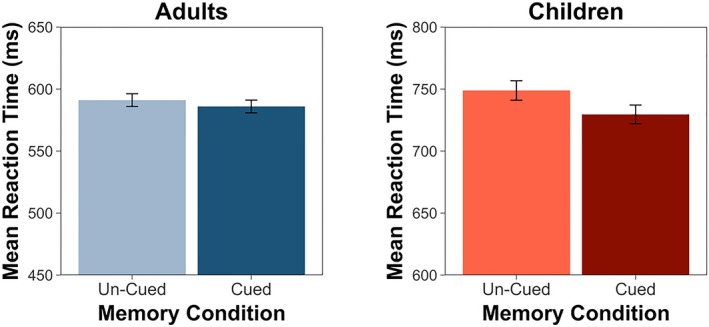
Experiment 3. While adults demonstrated no effect of memory condition on orienting reaction times, children responded more quickly on cued relative to un‐cued trials. Error bars indicate standard error. [Color figure can be viewed at wileyonlinelibrary.com]

We next analyzed our reaction‐time data. Our linear mixed‐effects model for reaction time included our fixed effects of interest (memory condition with two levels: cued and un‐cued; age group with two levels: adults and children) and random intercepts for subject and scene. As with our accuracy data, we observed a main effect of age group, with children (*M* = 739.5 ms, *SD* = 201.9 ms) responding more slowly across conditions than adults (*M* = 588.6 ms, *SD* = 163.2 ms), *F*(1, 50.1) = 32.0, *p *<* *.0001. We also observed a main effect of memory condition, such that participants responded more quickly on cued (*M* = 642.9 ms, *SD* = 189.9 ms) relative to un‐cued trials (*M* = 655.6 ms, *SD* = 198.6 ms), *F*(1, 3,295.1) = 7.49, *p* = .006. However, this effect was qualified by a Memory Condition × Age Group interaction effect, *F*(1, 3,295.2) = 4.07, *p* = .04 (Figure 4). To interpret this interaction, we ran separate models for adults and children. Including memory condition improved model fit for our child model only, such that memories that cued the target location facilitated faster orienting reaction times in children, *F*(1, 1,309.3) = 7.81, *p* = .005. Adults did not demonstrate an effect of memory condition on reaction times during orienting, *F*(1, 1,942.3) = 0.39, *p* = .53. These analyses indicate that, despite forming similarly precise memories for the target locations, children's attention orienting was influenced by these memories while adults’ orienting was not.

### Experiment 3 Discussion

This study aimed to extend our findings from Experiment 1 by comparing how memories influenced attention orienting across children and adults, in the absence of competing effects of exogenous cues. Previously, we found that when salient visual events occurred during orienting, children used memories to orient their attention while adults did not. However, in Experiment 2, we observed that adults *did* use memories to orient their attention when no salient visual events were present. Thus we hypothesized that the developmental differences we observed in Experiment 1 were primarily due to children and adults responding differently to the presence of the salient events. Without the source of exogenous orienting, we had hypothesized that adults would demonstrate a *stronger* influence of memory on attention, because the cognitive control mechanisms in the prefrontal cortex implicated in the coordination of attentional control and memory systems (Rosen et al., 2016; Stokes et al., 2012; Summerfield et al., 2006) continue to develop throughout the school‐aged years (Johnson et al., 2014). However, contrary to these predictions, we found that children demonstrated a *stronger* effect of memories on attention orienting. When cued by scenes with associated memory traces, children, but not adults, responded more quickly to targets presented in cued relative to un‐cued locations.

Our failure to observe an effect of memories on attention‐orienting consistently in adults is puzzling given that many previous studies of adults using similar tasks have found enhanced target sensitivity and faster target reaction times on memory‐cued relative to un‐cued trials (Patai et al., 2012; Salvato et al., 2016; Stokes et al., 2012; Summerfield et al., 2006, 2011). It may be the case that aspects of our task design that differ from previous studies (i.e., using a discrimination rather than a detection task; using a slightly larger orienting target; incorporating a potentially distracting, child‐friendly narrative) prevented adults’ from using memories to guide attention here. However, this is unlikely because we used a nearly identical design in Experiment 2, in which we *did* observe the expected effects.

Though further studies should attempt to replicate our findings, our data suggest that the effects of memories on attention orienting are more robust in children than adults, such that they emerge consistently under different task conditions and even when other sources of orienting are present. There are several possible reasons why this may be the case. First, previous studies have found that individuals use both exogenous and endogenous cues to a greater degree when orienting tasks are more difficult (Berger et al., 2005; Snowden, Willey, & Muir, 2001). For example, Berger et al. (2005) found that when a discrimination task was more difficult, participants demonstrated a larger endogenous cuing effect. Our accuracy and reaction‐time data suggest that children found our orienting task more difficult than adults. In Experiments 1 and 3, for example, children's reaction times were on average about 200 ms slower than adults’. Thus, it may simply be the case that children relied more on orienting cues than adults to perform the task, which they likely found more difficult. Future studies could probe the influence of task difficulty on the use of memory cues by manipulating difficulty within subject or within age group. That said, it is important to note that adults *did not* reach ceiling performance, with an average accuracy of only 86%. Additionally, in both Experiments 1 and 2, adults’ orienting reaction times were affected by the salient visual events, suggesting that the task was not so easy as to obscure any effects of cue‐driven modulations of attention.

## General Discussion

Across three experiments, we examined how memories and visual events differentially influence attention orienting in children and adults. We found that when both memory cues and visual events preceded a target in an orienting task, children demonstrated additive cuing benefits from both sources, whereas adults showed only exogenous cuing effects. Adults failed to use memories to guide attention when either predictive visual events or nonpredictive distractors appeared, suggesting that the sudden onset of peripheral, salient stimuli disrupted either the processes underlying memory recall or the use of memory to orient attention in adults but not children. Furthermore, adults use of memories to guide attention when no visual events occurred was inconsistent—emerging in one group of subjects but not another in a nearly identical task.

Our findings bolster previous claims of children's ability to use learned environmental regularities to shape their behavior (e.g., Dixon et al., 2010). Previous studies elucidated these effects through contextual cuing paradigms. Here, we provide the first evidence that scene‐based memory‐guided attention orienting is also intact in school‐aged children. Together with past work, our results suggest that the medial temporal lobe memory systems, fronto‐parietal orienting network, and prefrontal cognitive control systems that are implicated in memory‐guided attention (Goldfarb et al., 2016; Rosen et al., 2015; Stokes et al., 2012; Summerfield et al., 2006) are sufficiently mature in 7‐ to 12‐year‐olds to enable the use of past experience to guide attention‐orienting. This is consistent with work that suggests that the more posterior nodes of the cognitive control network located within the parietal lobe may play a particularly important role in coordinating the use of memory to guide attention (Rosen et al., 2015). These cortical areas reach maturity significantly earlier than the prefrontal nodes of the cognitive control network (Shaw et al., 2008), and may support memory‐guided attention in children. However, we also measured striking age‐related differences. If memory guides attention even in the presence of visual events in childhood, the obvious question then, is why does this relationship differ in adults? Though Experiment 2 ruled out differences in strategy use across children and adults, several other possible explanations persist.

One potential explanation for our findings is that the *time course* of the recruitment of the mechanisms underlying attention‐orienting changes over development. Past work has revealed that children are slower to disengage attention from a cued location and to reorient toward a new location (Schul et al., 2003). In our task, adults may have been able to modulate their attention in response to the initial presentation of the scene with the associated memory, and then rapidly disengage from it and shift their attention to the visual event prior to target onset. Even in the blocks when no separate visual event preceded the target, the onset of the target itself may have engaged exogenous orienting mechanisms. Children, however, may have lingered on the memory, unable to fully disengage and reorient to the visual event, such that when the target was presented, they were more successful in monitoring *both* cued locations relative to the adults. Thus children's inefficiency in orienting attention may actually be advantageous in situations when contextual information competes with the rapid onset of novel stimuli, particularly if those novel stimuli are irrelevant or distracting. Future studies should manipulate cue order and cue timing to better differentiate whether the cuing differences we observed across age groups are due specifically to changes in the use of memories to guide attention or a consequence of greater orienting efficiency with age.

It is also possible that children simply rely on past experiences to orient their attention to a greater degree than adults. Though past work has suggested that memory‐guided attention is less robust in children (Couperus et al., 2011; Vaidya et al., 2007), and that the neural systems supporting cognitive control and episodic memory are still developing throughout middle childhood (Ghetti & Bunge, 2012; Johnson et al., 2014), very few studies have directly compared memory‐guided attention in these two age groups using the same task. It is worth noting again that previous studies of memory‐guided attention in children have relied on contextual cuing tasks in which learning is implicit. Our use of scene cues could have engaged implicit learning, explicit memory representations, or some combination of both processes, potentially leading to different effects of previous experience on attention across development. Additionally, in our task, children completed a lengthy learning phase such that their memories for the target locations were comparable to those of adults; previous work in the domain of contextual cuing has suggested that children might not use learned regularities to guide attention because they fail to learn them in the first place (e.g., Couperus et al., 2011).

Although further work is needed to replicate our finding that children demonstrate larger effects of memories on attention relative to adults, it may be the case that the relative immaturity of children's memory systems may lead to *greater* use of memories to orient attention. Children demonstrate less flexibility in their retrieval of episodic memories (DeMaster, Coughlin, & Ghetti, 2016; Levy‐Gigi & Vakil, 2010; Townsend, Richmond, Vogel‐Farley, & Thomas, 2010). For example, DeMaster et al. (2016) had 8‐year‐olds, 10‐year‐olds, and young adults complete a task in which they encoded pairs of objects. At retrieval, the positions of some of the old object pairs were flipped, such that the item that was previously above was now presented below the other item. These change in positions did not affect memory performance in 10‐year‐olds or young adults, but 8‐year‐olds demonstrated significantly better memory performance when the items were presented in their original positions, suggesting that younger children's memory retrieval may be particularly impaired by contextual changes. In our task then, it may be the case that adults’ representations of the initial scene and target star were more flexible, such that the scene and its associated location were less strongly bound. Thus, when the scene later appeared at orienting, adults may not have been as impaired at identifying objects presented in new, un‐cued locations. In other words, children's more rigid representations of episodic information may lead to stronger effects of contextual information on future behavior, though much more work is needed to probe this possibility. One simple starting point could be to have the same participants complete a contextual memory task and a memory‐guided orienting task to determine if participants most impaired by contextual changes at retrieval also demonstrate the strongest cuing effects of memories on attention.

Interestingly, work with elderly adults (Salvato et al., 2016) suggests that the relationship between explicit memory performance and memory‐guided attention may also differ across age groups. Salvato et al. (2016) found that older adults demonstrated significantly worse explicit memories for locations in scenes relative to younger adults, and yet demonstrated equivalent facilitation of attention orienting when targets were presented in experienced locations. Future work should continue to probe the relationships between learning, explicit memory, and memory‐guided attention across the entire lifespan.

Though the neural systems underlying memory‐guided attention are generally thought to be complex and slow to mature, our findings suggest that memories may play a particularly important role in shaping attention early in life. In fact, many other sources of orienting, including internal (endogenous) goals and social stimuli, may *rely* on associations that children learn through experience over time. Furthermore, we found that memories guide attention even in the presence of salient, visual events. The ability to use memories to orient attention in the face of salient visual events may be a useful mechanism for suppressing distraction. Thus, we might expect that individuals who show greater use of memories to guide attention also demonstrate an increased ability to navigate and learn in familiar environments with frequent, transient distractors. Although our study was not designed a priori to investigate these individual differences, in either children or adults, and therefore it was not adequately powered to test them explicitly, future work could focus on testing this hypothesis in a large‐scale study focused on individual differences.

The role of memories in facilitating distractor suppression may have important consequences in real‐world learning settings. Although more work is needed to elucidate the influence of memories on attention in naturalistic contexts, our data suggest that familiar settings could potentially moderate the negative influence of visual distraction in children's environments. For example, children who have learned through experience to orient toward their classroom's chalkboard may be less likely to fully shift their attention toward a disruptive classmate on the other side of the room.

Many previous studies have found that across development, the engagement of visual attention influences learning and memory (Amso & Scerif, 2015; Astle, Nobre, & Scerif, 2012; Astle & Scerif, 2011; Markant & Amso, 2013, 2014, 2015; Doherty, Patai, Duta, Nobre, & Scerif, 2017; Shimi et al., 2014). Our findings bolster support for the idea that attention and memory interact reciprocally—attention shapes the memories we form, which in turn influence our patterns of attention, perhaps robustly during childhood. Thus, early differences in memory ability may contribute to early differences in attention, which in turn may affect future learning. Given the reciprocal interactions between memory and attention throughout development, any deficits in either of these processes is likely to affect the other, pointing to the importance of intervening early to prevent cascading learning problems.

## Data Availability

All data and analysis code pertaining to this article is available online: https://osf.io/fjpcg/.

## Supporting information


**Appendix S1.** Standardized (*Z*‐Scored) Reaction Time Analyses
