# Supplementary material for: Differential Effects of Salient Visual Events on Memory‐Guided Attention in Adults and Children
Source: Child Dev. 2018 Oct 8;90(4):1369–88. doi: 10.1111/cdev.13149 (PMC6767380; doi:10.1111/cdev.13149)
Supplement: Supplementary file 1 — Appendix S1. Standardized (Z‐Scored) Reaction Time Analyses [file CDEV-90-1369-s001.docx]

**Standardized (Z-Scored) Reaction Time Analyses**

We re-ran all the reaction time analyses in the paper using standardized reaction times for each participant rather than raw reaction time data. Results of these analyses are described for each experiment. As described in the paper, we followed the forward-stepping-best-path approach recommended in Barr, Levy, Scheepers, and Tily (2013) to determine the random effects structure of each model. All data and analysis code are available at osf.io/fjpcg/.

**Experiment 1**

**Effects of visual events on attention orienting.** We ran a linear mixed effect model to determine whether participants’ z-scored reaction times varied in response to the visual events. Our model included fixed effects of age group and visual event condition, random intercepts for subject and scene, and random slopes across visual event conditions for each subject and each scene. We observed a main effect of visual event condition, *F*(2, 62.96) = 11.33, *p* < .001. Follow-up paired comparisons with Tukey contrast indicated that participants responded significantly more slowly to un-cued targets relative to those in the cued (*p* < .001) and neutral (*p* = .014) condition. There was no difference in standardized reaction times between the cued and neutral conditions (*p* = .076). Furthermore, there was no effect of age group on participants’ standardized reaction times, *F*(1, 48.69) = .03, *p* = .86, nor was there a visual event condition x age group interaction effect, *F*(2, 47.76) = 1.1, *p* = .34.

**Interactions between memory cues and visual events on attention orienting.** To examine the effects of memory cues and salient visual events on attention orienting, we ran a linear mixed effect model examining participants’ z-scored reaction times. The model included fixed effects of age group, memory condition and visual event condition, random intercepts for subject and scene, and random slopes across memory conditions for each scene. Results aligned with our previous analysis of raw reaction time data: We observed a main effect of visual event condition, *F*(1, 3351.43) = 78.71, *p* < .0001, with participants responding faster on trials cued by visual events. We also observed a main effect of memory condition, *F*(1, 158.92) = 3.83, *p* = .05. This effect was qualified by an age group x memory condition interaction effect, *F*(1, 3271.47) = 5.81, *p* = .02.

As with our analysis of the raw reaction time data, separate models for children and adults indicated that children responded more quickly to targets cued by memories, *F*(1, 1417.53) = 8.39, *p* = .004, but adults did not, *F*(1, 1900.92) = .04, *p* = .84.

**Experiment 2**

**Effects of memories on attention orienting.** We ran a linear mixed effect model to determine whether participants’ z-scored reaction times varied as a function of memory cue condition. Our final model included a fixed effect of memory cue condition, random intercepts for subject and scene, and random slopes across memory cue conditions for each scene. Unlike our analysis of raw reaction times, there was not a significant effect of memory condition on participants z-scored reaction times, *F*(1, 146.57) = 3.13, *p* = .08.

**Effects of visual distractors on attention orienting.** To examine the effects of visual distractors on z-scored reaction times, we ran a linear mixed effect model with distractor location as a fixed effect, subject and scene as random intercepts, and allowed for different slopes for each subject across distractor locations. The model indicated a main effect of distractor location, *F*(2, 25.55) = 7.61, *p* = .003. Post-hoc Tukey contrasts indicated that participants responded significantly faster on no-distractor trials relative to trials with both near and far distractors (*p*s <.03). There was no difference in participants’ reaction times between near and far-distractor trials (*p* = .93).

**Interactions between memories and visual events on attention orienting.** We ran a linear mixed effect model to determine whether participants’ z-scored reaction times varied as a function of memory cue condition and distractor location. Our final model included fixed effects for memory condition and distractor location and random intercepts for subject and scene. There was no effect of memory condition or distractor location, nor was there an interaction between them on participants’ z-scored reaction times, all *p*s > .50.

**Experiment 3**

**Effects of memories on attention orienting.** We ran linear mixed effect model to determine whether participants’ z-scored reaction times varied as a function of memory cue condition. Our final model included fixed effects of memory cue condition and age group and random intercepts for subject and scene. Participants responded significantly faster on cued relative to un-cued trials, *F*(1, 3318.0) = 7.58, *p* = .006. Unlike our analysis of raw reaction times, there was not a significant age group x memory cue condition interaction effect, *F*(1, 3318.6) = 3.01, *p =* .08.

However, given our a priori hypothesis that the effects of memories on attention may differ across age groups, we still ran separate models for children and adults. As with our analysis of the raw reaction time data, here we observed that children responded more quickly on to targets cued by memories relative to un-cued targets, *F*(1, 1319.2) = 8.45, *p* = .004, but adults did not, *F*(1, 1949.72) = .66, *p* = .42.
